# Short-term BMI trajectories as a prognostic predictor in patients with pancreatic cancer

**DOI:** 10.3389/fnut.2025.1680626

**Published:** 2026-01-30

**Authors:** Chenxi Li, Yan Zhong, Wenying Wang, Xin Jin, Xiaona Wang

**Affiliations:** 1 Senior Department of Oncology, Chinese PLA General Hospital, Beijing, China; 2 Department of Radiology, The Fourth Medical Center, Chinese PLA General Hospital, Beijing, China; 3 Senior Department of Obstetrics & Gynecology, The Seventh Medical Center, Chinese PLA General Hospital, Beijing, China; 4 Senior Department of Hepato- Pancreato- Biliary Surgery, The First Medical Center, Chinese PLA General Hospital, Beijing, China; 5 Department of Cardiology, The Second Medical Center & National Clinical Research Center for Geriatric Diseases, Chinese PLA General Hospital, Beijing, China

**Keywords:** BMI trajectories, marker, overall survival, pancreatic cancer, prognosis

## Abstract

**Objective:**

While numerous studies have examined the association between body mass index (BMI) and cancer prognosis, most have only captured BMI at a single time point. Whether BMI trajectories are linked to the prognosis of pancreatic cancer remains unclear. This study aimed to investigate the relationship between short-term BMI trajectories and clinical outcomes in patients with pancreatic cancer.

**Methods:**

A retrospective study was performed on 200 pancreatic cancer patients who admitted to our hospital from January 1, 2017 to December 31, 2021. BMI 1 (BMI of patients diagnosed with pancreatic cancer 1 year before), BMI 2 (BMI of the patient at diagnosis of pancreatic cancer), BMI 3 (BMI of patients 6 months after diagnosis of pancreatic cancer), BMI 4 (BMI 1–BMI 2), BMI 5 (BMI 3–BMI 2) and BMI 6 (the longitudinal BMI trajectory) were recorded. Clinical-pathological characteristics, oncologic outcomes, progression free survival (PFS) and overall survival (OS) were collected. The prognostic significance was determined by Kaplan-Meier analysis, Cox proportional hazards and restricted cubic spline regression models.

**Results:**

We found that changes in BMI may be a predictor of pancreatic cancer survival. The results of the multivariate analysis of factors influencing the pancreatic cancer OS showed that BMI 4 ≥1.3 (HR: 1.69, 95% CI: 1.19–2.41, *P* = 0.004), BMI 5 <−0.3 (HR: 3.05, 95% CI: 2.01–4.65, *P* < 0.001) and BMI 6 (patients from normal BMI to low BMI, HR: 2.51, 95% CI: 1.09–5.77, *P* = 0.031; patients from high BMI to normal BMI, HR: 2.01, 95% CI: 1.09–3.70, *P* = 0.025) indicated higher mortality rate.

**Conclusion:**

This study confirms that short-term BMI trajectories before and after diagnosis, as well as early during treatment, are independent prognostic factors for both overall survival and progression-free survival in pancreatic cancer patients. Particular attention should be paid to patients who are normal-weight at diagnosis but transition to a low-BMI category shortly after treatment, as they face the highest mortality risk.

## Introduction

Pancreatic cancer (PC), known as the “king of cancer,” has become a major problem in the medical field because of its hidden onset, low early diagnosis rate, rapid progression and poor prognosis. This disease has a poor prognosis and is ranked as the sixth leading cause of cancer mortality in both sexes combined, accounting for almost 5% of all cancer deaths worldwide (1). The global 5-year survival rate for pancreatic cancer has shown minimal improvement over the past few decades. In 2019, the 5-year survival rate for pancreatic cancer in China was only 7.2%, while it was 9% in the United States and Europe. However, by 2022, the survival rate had increased to 11% in the United States and Europe, and further improved to 12% by 2023 (2). Improving the survival rate of PC patients and achieving early screening and diagnosis have always been key focuses for medical experts. Therefore, in our clinical practice, it is crucial to remain highly vigilant and pay close attention to a range of related clinical manifestations such as weight loss, jaundice, and pain to provide early diagnosis and intervention treatment.

In the past few decades, the number of obese and overweight patients has increased, and the risk of tumor and metabolic diseases brought by weight gain has greatly increased. There are evidences showing that nutritional status, serum lipids, and Inflammatory index relates to oncological outcomes of different types of cancer (3–5). Body mass index (BMI) is a simple and commonly used indicator for evaluating the nutritional status in clinical practice. Some epidemiological studies have investigated the relationship between BMI and pancreatic cancer, but the conclusions are inconsistent and only record BMI as an indicator at a single point in time (6–11). Changes in body weight over time may be relevant to the etiology of pancreatic cancer. In clinical practice, we have found that many patients with pancreatic cancer experience weight loss. Numerous studies have shown that weight loss is associated with a poor prognosis of tumors. However, there are currently few studies that have recorded the changes in BMI of pancreatic cancer patients, and further explored whether the changes in BMI trajectories are related to the prognosis of pancreatic cancer. Therefore, the relationship between BMI trajectories and prognosis of PC needs further clarification.

**Figure 1:**
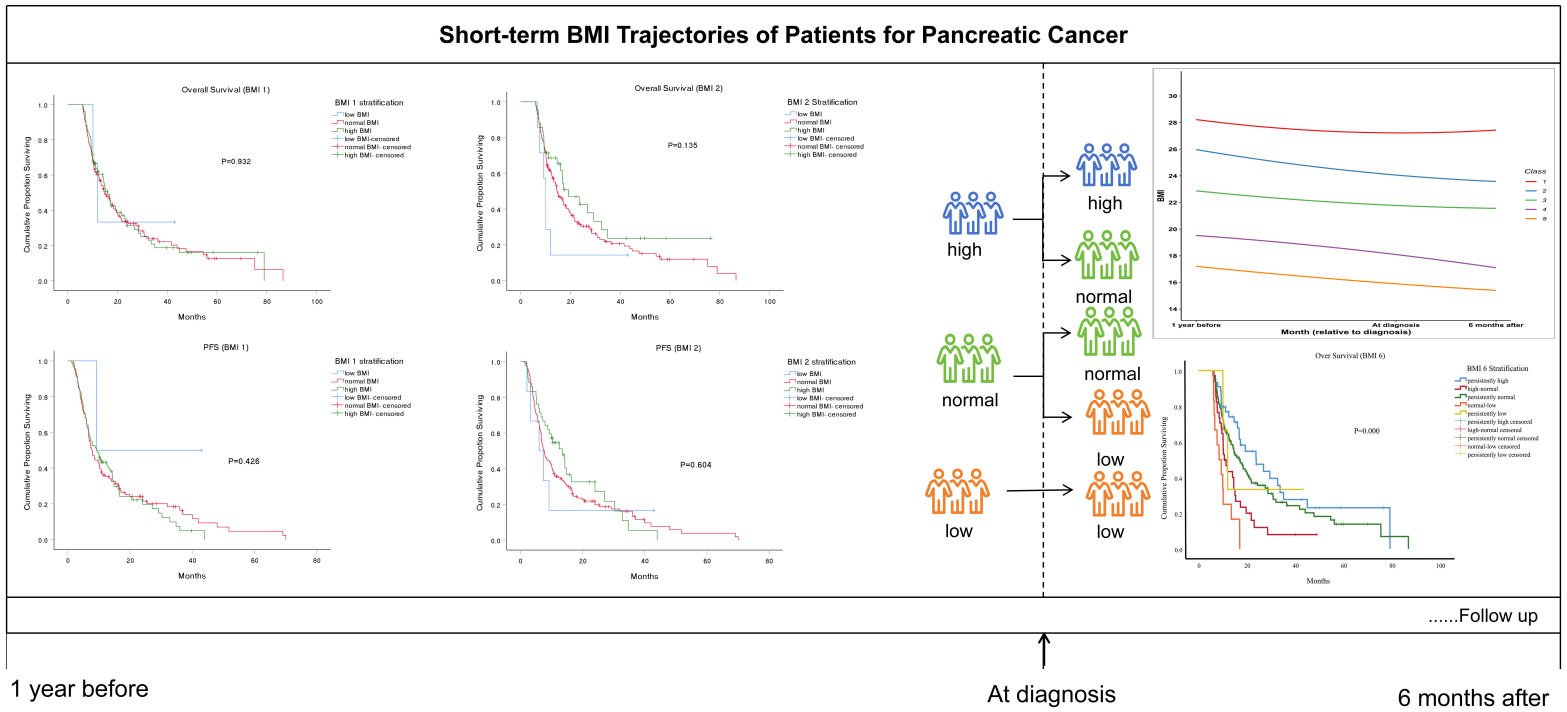
Workflow.

To bridge this knowledge gap, we conducted a retrospective analysis, focusing on the BMI measured at the time of pancreatic cancer diagnosis, 1 year before diagnosis, 6 months after diagnosis, and the longitudinal trajectory of BMI. We explored the relationship between these factors and the prognosis of pancreatic cancer (Figure 1).

## Methods

### Study design and patients

This was a retrospective study. The study included patients diagnosed with pancreatic cancer who were admitted to the Fourth Medical Center of Chinese PLA General Hospital between January 1, 2017 to December 31, 2021. The inclusion criteria were: (1) > 18 year; (2) pathologically diagnosed with pancreatic cancer. The exclusion criteria were: (1) Patients with serious cardio-cerebral vascular disease, kidney disease, liver disease, obvious ascites and edema or hematopoietic disease (15 cases). (2) incomplete information (45 cases). (3) loss to follow-up (30 cases). Finally, a total of 200 patients with pancreatic cancer were included in this study. According to NCCN guidelines, patients who have not undergone surgery are diagnosed and staged using computed tomography (CT) and/or magnetic resonance imaging (MRI) of the pancreas and biopsy. Patients who have undergone surgery can be staged according to postoperative pathology. The Medical Ethics Committee of Center of PLA General Hospital has reviewed and approved this project, and this were followed in accordance with relevant laws, Declaration of Helsinki and other ethical principle.

### Data collection

The clinical and pathological information of all enrolled patients was obtained by the electronic medical record system. All body weight measurements were taken in the morning on an empty stomach, and the staff responsible for the measurements were uniformly trained and used the same electronic measurement tools to ensure accuracy as much as possible. Patients without complete information were followed up by telephone to supplement the information data. Progression-free survival (PFS) and overall survival (OS) were obtained. PFS is understood as the time from the first day of surgery or chemotherapy date to the date of disease progression. Disease progression is determined by the clinician and radiologist together. PFS was also defined as the date of death or the last follow-up. OS is described as the time from the first day of the date of surgery or chemotherapy, to the date of death or the last follow-up. Follow-up was ended on January 31st, 2023.

### Grouping

Wasting is one of the clinical features of pancreatic cancer. Patients diagnosed with pancreatic cancer or undergo pancreatic cancer surgery, BMI will show varying degrees of decline, some patients after receiving effective treatment or nutritional support, weight recovery. We investigated the BMI trajectories of enrolled patients before and after diagnosis of pancreatic cancer.

Our study investigated the BMI of enrolled patients at 3 time points and further explored the relationship between BMI and pancreatic cancer prognosis.

① BMI 1: BMI 1: BMI 1 year before diagnosis.

② BMI 2: BMI at diagnosis.

③ BMI 3: BMI 6 months after diagnosis.

④ BMI 4 = BMI 1—BMI 2 (change in BMI from 1 year before diagnosis to diagnosis).

⑤ BMI 5 =BMI 3—BMI 2 (change after anti-tumor therapy).

⑥ BMI 6: longitudinal trajectories based on BMI 2 and BMI 3, categorized into: (1) Persistently high; (2) High to normal; (3) Persistently normal; (4) Normal to low; (5) Persistently low.

**Figure 2:**
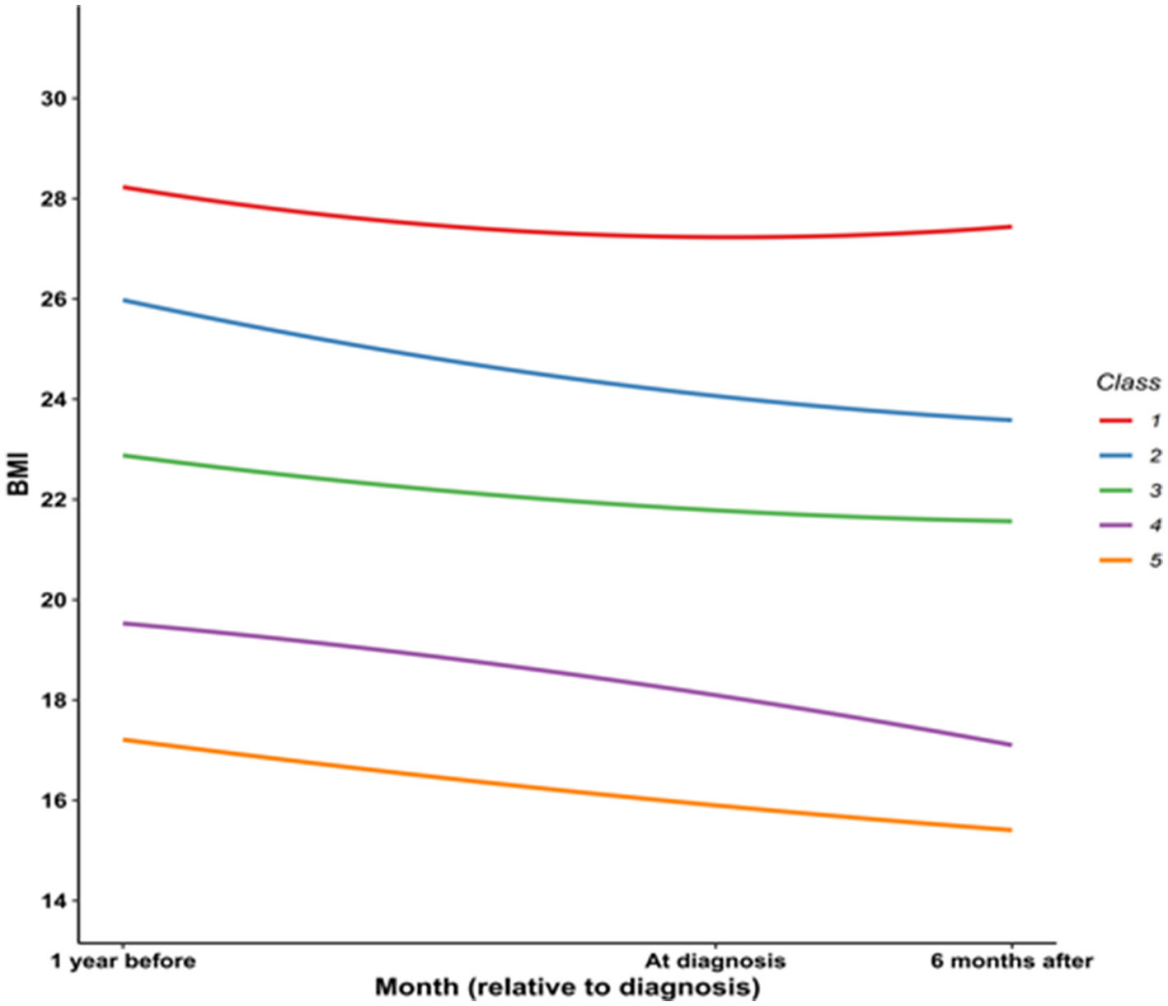
The trajectories in BMI of 200 patients over a period of one and a half years before and after they were diagnosed with pancreatic cancer.

Figure 2 shows the trajectories in BMI of 200 patients over a period of one and a half years before and after they were diagnosed with pancreatic cancer. The weight of most patients shows a downward trend. After anti-tumor treatment, the weight of a small number of patients can gradually return.

Individuals were divided into the three weight status groups: normal weight group (BMI: 18–24.9 kg/m^2^), underweight (BMI: <18 kg/m^2^), overweight and obesity (BMI: ≥25 kg/m^2^). The median is used as the grouping basis for BMI 4 and BMI 5.

### Statistical analysis

According to the test report provided by the laboratory, the low and high reference values were 7.0 mmol/L for fast blood glucose, 27 U/ml for CA199.

SPSS version 26.0 software and R software (version 4.4.0) were used for statistical analysis. Descriptive analyses or continuous variables were presented as mean ± standard deviation (x ± s), and categorical variables were described as percentages (%). The OS curve and PFS curve of each group were plotted Kaplan-Meier's method. Potential short–and long-term prognostic factors of pancreatic cancer were explored through univariate and multivariable analysis using a Cox proportional hazards regression model. Three models were established, respectively. Model 1 did not adjust for covariates, Model 2 adjusted for gender and age, and Model 3 adjusted for gender, age, drinking history, smoking history, history of cancer, T stage, N stage, TNM stage, tumor location, differentiation, CA199, fast blood glucose and surgery. The hazard ratio (HR) and its 95% confidence interval (CI) were calculated for all. The association between clinicopathological factors of the patients and BMI 5 levels was compared with the chi-square test and Fisher's exact test. Furthermore, restricted cubic splines (RCSs) were used to elucidate the relationships between BMI and overall survival. *P* value <0.05 was considered statistically significant in all analyses.

## Results

### Characteristics of the patients

From January 1, 2017 to December 31, 2021, 290 patients were admitted for pancreatic cancer. 90 people were excluded because of missing data or suffering from other serious illnesses. Finally, 200 patients were included, of whom 147 were deceased, and 53 were alive. The demographic characteristics of patients are shown in Table 1. Most of the subjects were older than 60 years, and 62.5% were male. Consistent with previous reports, most of the tumors were located in the head of the pancreas (60.5%). 174 cases (87%) tumor tissues were moderate-poorly differentiated, and only 26 (13%) cases of high differentiation. The number of TNM classification of patients in this study was as follows: 44 patients in stage I, 47 patients in stage II, 44 patients in stage III, 65 patients in stage IV. The median values for BMI 4 and BMI 5 are 1.3 and −0.3, respectively. Only three patients (1.5%) had a low BMI (<18 Kg/m^2^) before pancreatic cancer diagnosis, but this proportion increased significantly (14 cases, 7%) 6 months after pancreatic cancer diagnosis.

**Table 1:** Clinicopathological characteristics in the population.

**Characteristics**	**Patients**	**%**
**Age (year)**		
<60	74	37.0%
≥60	126	63.0%
**Sex**		
Male	125	62.5%
Female	75	37.5%
**Tumor location**		
Pancreatic head	121	60.5%
Pancreas body and tail	79	39.5%
**T stage**		
1	10	5.0%
2	72	36.0%
3	55	27.5%
4	63	31.5%
**N stage**		
0	107	53.5%
1	48	24.0%
2	45	22.5%
**M stage**		
0	135	67.5%
1	65	32.5%
**TNM stage**		
1	44	22.0%
2	47	23.5%
3	44	22.0%
4	65	32.5%
**Family history**		
Yes	40	20.0%
No	160	80.0%
**Tumor differentiation degree**		
Well	26	13.0%
Moderate	105	52.5%
Poor	69	34.5%
**Smoke**		
Yes	86	43.0%
No	114	57.0%
**Alcohol**		
Yes	71	35.5%
No	129	64.5%
**CA199**		
<27 U/ml	35	17.5%
≥27 U/ml	165	82.5%
**Fast blood glucose (mmol/L)**		
<7.0	132	66.0%
≥7.0	68	34.0%
**BMI 1 (Kg/m** ^2^ **)**		
<18	3	1.5%
18–25	119	59.5%
≥25	78	39.0%
**BMI 2 (Kg/m** ^2^ **)**		
<18	7	3.5%
18–25	151	75.5%
≥25	42	21.0%
**BMI 3 (Kg/m** ^2^ **)**		
<18	14	7.0%
18–25	142	71.0%
≥25	44	22.0%

BMI 1: BMI of patients diagnosed with pancreatic cancer 1 year before. BMI 2: BMI of the patient at diagnosis of pancreatic cancer. BMI 3: BMI of patients 6 months after diagnosis of pancreatic cancer.

### Factors associated with PFS

In this study, we analyzed the effect of BMI on PFS at different time points. Using univariate Cox proportional hazards model (Table 2) found that the factors associated with the pancreatic cancer PFS were the BMI 3 (*P* < 0.001), BMI 4 (HR: 1.60, 95% CI: 1.14–2.15, *P* = 0.006), BMI 5 (HR: 2.55, 95% CI: 1.84–3.52, *P* < 0.001) and BMI 6 (*P* < 0.001). The Kaplan-Meier curve provides a more intuitive illustration of the relationship between BMI and PFS (Supplementary Figure 1). In order to determine the independent prognostic factors, the significant variables from univariate analysis were further subjected to multivariate regression analyses. As shown in Table 2, BMI 4 ≥1.3 (HR: 1.52, 95% CI: 1.08–2.13, *P* = 0.016); BMI 5 <−0.3 (HR: 2.45, 95% CI: 1.66–3.61, *P* < 0.001) and BMI 6 (patients from normal BMI to low BMI, HR: 3.10, 95% CI: 1.44–6.69, *P* = 0.004; patients from high BMI to normal BMI, HR: 1.72, 95% CI: 0.99–2.98, *P* = 0.054) were related with poor PFS. On the contrary, normal and overweight of BMI 3 (HR: 0.46, 95% CI: 0.24–0.89, *P* = 0.022; HR: 0.40, 95% CI: 0.19–0.84, *P* = 0.016, respectively) was related with longer PFS. Forest plot (Supplementary Figure 2) more intuitively demonstrate the influence of different factors on PFS. TNM stage and BMI 6 have predictive effects on PFS.

**Table 2:** Univariate and multivariate cox proportional hazard model of pancreatic cancer with PFS.

	**Model 1**		**Model 2**		**Model 3**	
**BMI**	**Hazard ratio (95% CI)**	***P*** **-value**	**Hazard ratio (95% CI)**	***P*** **-value**	**Hazard ratio (95% CI)**	***P*** **-value**
BMI 1 (Kg/m^2^)		0.467		0.521		0.602
<18	1		1		1	
18–25	3.17 (0.44–22.89)	0.252	2.95 (0.41–21.36)	0.284	2.53 (0.33–19.33)	0.371
≥25	3.38 (0.47–24.55)	0.228	3.12 (0.43–22.73)	0.262	2.71 (0.35–20.71)	0.337
BMI 2 (Kg/m^2^)		0.607		0.553		0.708
<18	1		1		1	
18–25	0.90 (0.37–2.22)	0.821	0.86 (0.35–2.12)	0.742	0.74 (0.28–1.96)	0.546
≥25	0.75 (0.29–1.93)	0.543	0.70 (0.27–1.82)	0.467	0.67 (0.25–1.82)	0.429
BMI 3 (Kg/m^2^)		<0.001		<0.001		0.046
<18	1		1		1	
18–25	0.29 (0.16–0.52)	<0.001	0.31 (0.17–0.57)	<0.001	0.46 (0.24–0.89)	0.022
≥25	0.23 (0.12–0.45)	<0.001	0.24 (0.13–0.48)	<0.001	0.40 (0.19–0.84)	0.016
BMI 4 (Kg/m^2^)		0.006		0.012		0.016
<1.3	1		1		1	
≥1.3	1.60 (1.14–2.15)		1.52 (1.10–2.10)		1.52 (1.08–2.13)	
BMI 5 (Kg/m^2^)		<0.001		<0.001		<0.001
≥−0.3	1		1		1	
<−0.3	2.55 (1.84–3.52)		2.65 (1.90–3.68)		2.45 (1.66–3.61)	
BMI 6 (Kg/m^2^)		<0.001		<0.001		0.013
Persistently high	1		1		1	
High-normal	1.96 (1.20–3.22)	0.007	2.06 (1.24–3.39)	0.005	1.72 (0.99–2.98)	0.054
Persistently normal	1.11 (0.74–1.66)	0.628	1.13 (0.76–1.70)	0.544	1.06 (0.68–1.66)	0.786
Normal-low	5.04 (2.55–9.98)	<0.001	4.67 (2.34–9.34)	<0.001	3.10 (1.44–6.69)	0.004
Persistently low	0.37 (0.05–2.75)	0.333	0.41 (0.06–3.00)	0.377	0.42 (0.06–3.29)	0.412

Model 1 was unadjusted. Model 2 was adjusted for age, sex. Model 3 was adjusted for age, sex, TNM stage, tumor location, differentiation, smoke, family history, alcohol, CA199, fast blood, surgery.

### Factors associated with OS

During the follow-up period of this study, 147 of 200 patients (73.5%) died, with a mOS of 19.3 months. Death was more common in patients with BMI 3 <18 kg/m^2^ (92.8%, 13/14) compared with that in patients with higher BMI 3 (72.0%, 134/186, *P* < 0.001). Univariate analyses revealed that BMI 3, BMI 4, BMI 5 and BMI 6 were associated with OS (Table 3).

**Table 3:** Univariate and multivariate cox proportional hazard model of pancreatic cancer with OS.

	**Model 1**		**Model 2**		**Model 3**	
**BMI**	**Hazard ratio (95% CI)**	***P*** **-value**	**Hazard ratio (95% CI)**	***P*** **-value**	**Hazard ratio (95% CI)**	***P*** **-value**
BMI 1 (Kg/m^2^)		0.932		0.935		0.985
<18	1		1		1	
18–25	1.26 (0.31–5.11)	0.751	1.23 (0.30–5.02)	0.772	1.03 (0.23–4.51)	0.973
≥25	1.21 (0.94–4.95)	0.795	1.18 (0.29–4.87)	0.820	1.06 (0.24–4.65)	0.940
BMI 2 (Kg/m^2^)		0.142		0.152		0.183
<18	1		1		1	
18–25	0.56 (0.25–1.29)	0.173	0.56 (0.25–1.29)	0.173	0.49 (0.20–1.20)	0.118
≥25	0.42 (0.17–1.03)	0.059	0.43 (0.17–1.05)	0.062	0.41 (0.16–1.06)	0.065
BMI 3 (Kg/m^2^)		<0.001		<0.001		0.151
<18	1		1		1	
18–25	0.32 (0.18–0.59)	<0.001	0.35 (0.19–0.65)	0.001	0.63 (0.31–1.25)	0.187
≥25	0.21 (0.11–0.42)	<0.001	0.23 (0.12–0.47)	<0.001	0.46 (0.21–1.02)	0.055
BMI 4 (Kg/m^2^)		0.005		0.009		0.004
<1.3	1		1		1	
≥1.3	1.60 (1.15–2.22)		1.57 (1.12–2.20)		1.69 (1.19–2.41)	
BMI 5 (Kg/m^2^)		<0.001		<0.001		<0.001
≥−0.3	1		1		1	
<−0.3	3.22 (2.28–4.56)		3.24 (2.27–4.60)		3.05 (2.01–4.65)	
BMI 6 (Kg/m^2^)		<0.001		<0.001		0.050
Persistently high	1		1		1	
High-normal	2.42 (1.42–4.14)	0.001	2.36 (1.37–4.06)	0.002	2.01 (1.09–3.70)	0.025
Persistently normal	1.37 (0.88–2.13)	0.168	1.36 (0.87–2.14)	0.184	1.22 (0.74–2.02)	0.430
Normal-low	5.09 (2.47–10.49)	<0.001	4.61 (2.20–9.67)	<0.001	2.51 (1.09–5.77)	0.031
Persistently low	1.20 (0.28–5.06)	0.806	1.20 (0.28–5.10)	0.808	1.23 (0.28–5.55)	0.784

Model 1 was unadjusted. Model 2 was adjusted for age, sex. Model 3 was adjusted for age, sex, T stage, N stage, TNM stage, tumor location, differentiation, smoke, family history, alcohol, CA199, fast blood glucose, surgery.

**Figure 3:**
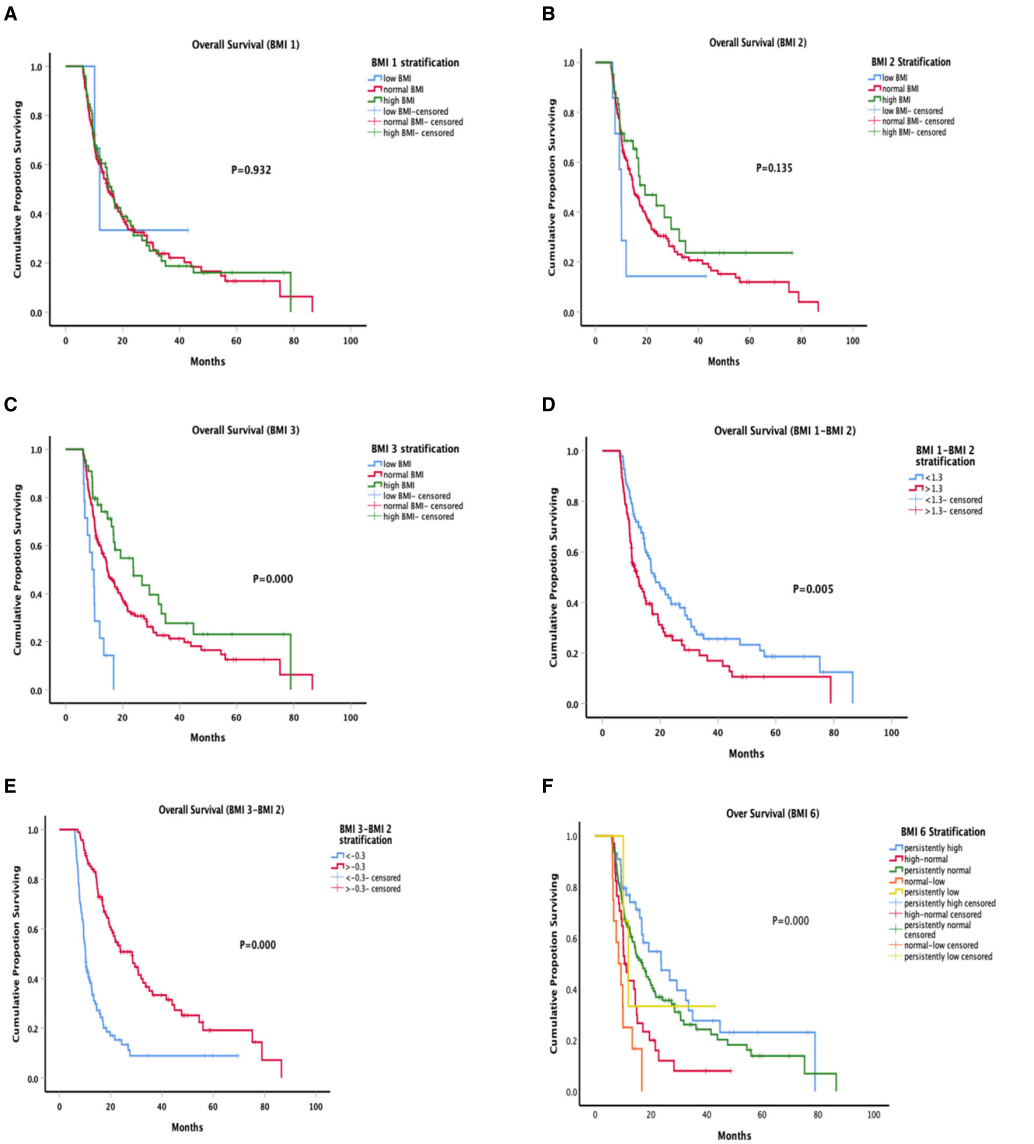
Kaplan-Meier survival curves according to different BMI measures. There were significant differences in BMI 3 **(C)**, BMI 4 **(D)**, BMI 5 **(E)**, and BMI 6 **(F)** (*P* < 0.001, *P* = 0.005, *P* < 0.001), no significant differences were observed at BMI 1 and BM 2.

The OS curves are shown in Figure 3 by BMI groups; No significant differences were observed in BMI 1 and BMI 2 stratified analyses (Figures 3A, B). In the Kaplan-Meier curve for BMI 3, the OS was shorter in the low BMI group than in the normal group and high group (Figure 3C), the mOS were 9.3 months, 14.5 months and 23.8 months, respectively (*P* < 0.001). In the Kaplan-Meier curve for BMI 4 (BMI 1-BMI 2), the 1-year OS rate was 71.6 vs. 45.7% in the two groups (Figure 3D, *P* = 0.005). BMI after anti-tumor therapy minus BMI at diagnosis was defined as BMI 5, and the median value of BMI 5 was −0.3 (Figure 3E). The OS was longer in the BMI > −0.3 group, statistically significant difference was observed in the stratified analyses (*P* < 0.001). The trajectory of BMI shows that those whose weight did not change significantly, especially those who maintained a high BMI or a normal BMI, had a longer overall survival (Figure 3F).

**Figure 4:**
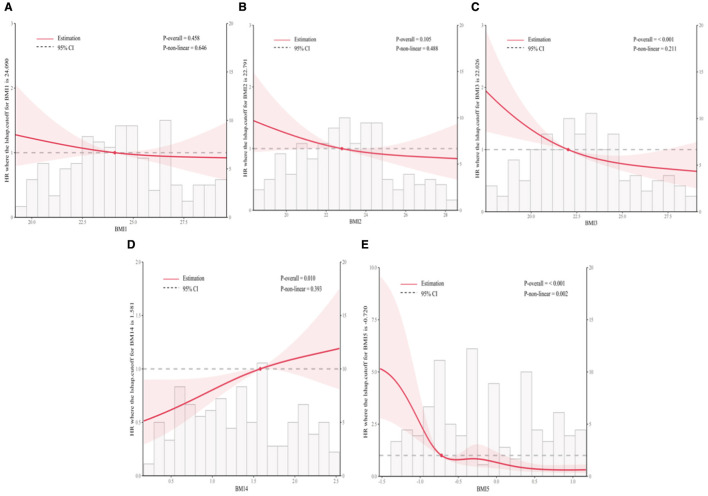
Restricted cubic spline analyses of the association between BMI measures and overall survival. There were significant differences in BMI 3 **(C)**, BMI 4 **(D)**, BMI 5 **(E)** (*P* < 0.001, *P* = 0.005, *P* < 0.001), no significant differences were observed at BMI 1 and BM 2.

Overall survival was linearly associated with the BMI 1, BMI 2, BMI 3 and BMI 4 according to RCS analysis (*P* values for nonlinearity of 0.646, 0.488, 0.211 and 0.393, respectively). However, a nonlinear relationship was observed between the BMI 5 and the OS, *P* = 0.002 (Figures 4A–E).

Furthermore, the results of the multivariate analysis of factors influencing the pancreatic cancer OS are presented in Table 3, BMI 4 ≥1.3 (HR: 1.69, 95% CI: 1.19–2.41, *P* = 0.004), BMI 5 <−0.3 (HR: 3.05, 95% CI: 2.01–4.65, *P* < 0.001) and BMI 6 (patients from normal BMI to low BMI, HR: 2.51, 95% CI: 1.09–5.77, *P* = 0.031; patients from high BMI to normal BMI, HR: 2.01, 95% CI: 1.09–3.70, *P* = 0.025) indicated higher mortality rate. Forest plot (Supplementary Figure 3) more intuitively demonstrate the influence of different factors on OS. TNM stage and BMI 6 have predictive effects on OS. So, we found that changes in BMI and BMI Trajectories may be predictor of pancreatic cancer survival.

The association between BMI changes (before and after antitumor therapy, BMI 5) and clinicopathological characteristics is summarized in Table 4. Of note, BMI 5 was significantly correlated with T stage (*P* = 0.025), lymph node stage (*P* = 0.001), TNM stage (*P* < 0.001), family history (*P* = 0.034), and CA 199 (*P* = 0.045). However, there was no significant relationships between BMI 5 and age, sex, tumor location, differentiation, smoke, alcohol, fast blood glucose.

**Table 4:** Clinical characteristics of 200 patients with different BMI changes after anti-tumor therapy.

**Characteristics**	**BMI 5 ≥−0.3 (%)**	**BMI 5 <−0.3 (%)**	***P*-value**
**Age (year)**			
<60	40 (42.1)	34 (32.4)	0.155
≥60	55 (57.9)	71 (67.6)	
**Sex**			
Male	62 (65.3)	63 (60.0)	0.443
Female	33 (34.7)	42 (40.0)	
**Tumor location**			
Pancreatic head	59 (62.1)	62 (59.0)	0.659
Pancreatic body and tail	36 (37.9)	43 (41.0)	
**Differentiation**			
Well	13 (13.7)	13 (12.4)	0.710
Moderate	52 (54.7)	53 (50.5)	
Poor	30 (31.6)	39 (37.1)	
**T stage**			
1	9 (9.5)	1 (1.0)	0.025
2	37 (38.9)	35 (33.3)	
3	23 (24.2)	32 (30.5)	
4	26 (27.4)	37 (35.2)	
**N stage**			
0	62 (65.3)	45 (42.9)	0.001
1	22 (23.2)	26 (24.8)	
2	11 (11.6)	34 (32.4)	
**M stage**			
0	78 (82.1)	57 (54.3)	<0.001
1	17 (17.9)	48 (45.7)	
**TNM stage**			
1	26 (27.4)	18 (17.1)	<0.001
2	29 (30.5)	18 (17.1)	
3	23 (24.2)	21 (20.0)	
4	17 (17.9)	48 (45.7)	
**Smoke**			
Yes	44 (46.3)	42 (40.0)	0.368
No	51 (53.7)	63 (60.0)	
**Family history**			
Yes	13 (13.7)	27 (25.7)	0.034
No	82 (86.3)	78 (74.3)	
**Alcohol**			
Yes	39 (41.1)	32 (30.5)	0.119
No	56 (58.9)	73 (69.5)	
<27	22 (23.2)	13 (12.4)	0.045
≥27	73 (76.8)	92 (87.6)	
**Fast blood glucose (mmol/L)**			
<7.0	57 (60.0)	75 (71.4)	0.088
≥7.0	38 (40.0)	30 (28.6)	

### Subgroup analyses

Forest plots of relationship between BMI 6 and PFS/OS in different subgroups of patients (Supplementary Figures 4, 5). Subgroup analyses were performed for surgery (yes or no), and no significant interactions were found in each subgroup (*P* = 0.723, *P* = 0.907, respectively). In each subgroup, weight loss–particularly the transition from normal to low BMI–was consistently associated with a poorer prognosis in patients with pancreatic cancer.

## Discussion

Our study identifies short-term BMI trajectories as an independent prognostic factor in pancreatic cancer, extending previous reports based solely on baseline BMI measurements. Specifically, rapid pre-diagnostic weight loss (BMI 4 ≥1.3 kg/m^2^), early post-treatment weight decline (BMI 5 <−0.3 kg/m^2^) and the longitudinal BMI trajectories–BMI 6 were strongly associated with shorter PFS and OS. On the contrary, those who maintained a high BMI or a normal BMI, had a longer overall survival. These dynamic changes appear to capture the combined effects of tumor progression, metabolic alterations, and treatment-related catabolism more accurately than static BMI values.

Weight loss in pancreatic cancer patients results from multiple interacting factors. Tumor-induced hypermetabolism and systemic inflammatory responses increase energy expenditure, while exocrine pancreatic insufficiency leads to malabsorption of fats and proteins, further limiting nutrient availability. Additionally, chemotherapy-related nausea, vomiting, and anorexia, as well as postoperative alterations in gastrointestinal anatomy, significantly compromise nutritional intake and absorption. This progressive nutritional depletion not only exacerbates muscle wasting and functional decline but also directly reduces patient tolerance to anti-tumor treatments such as surgery and chemotherapy.

Currently, the impact of weight loss on the prognosis of patients with pancreatic cancer has received more attention. The results of the meta-analysis by Wen et al. (12) show that underweight was associated with poorer overall survival (OS) in patients with digestive tract tumors when compared to those with normal body weight. Paccagnella et al. (13) have pointed out that weight loss is a non-specific clinical manifestation of tumor patients and is also an important predictor of death. It has a high incidence in a variety of digestive and non-digestive tract malignant tumors, such as esophageal cancer, gastric cancer, colorectal cancer, pancreatic cancer, and ovarian cancer. Compared with patients without weight loss, those with weight loss have significantly reduced sensitivity and tolerance to treatment, shortened survival time, and significantly reduced quality of life (14–16). Lakato et al. (17) pointed out that 63% of patients with pancreatic cancer had weight loss. Data about 1,020 patients with unresectable pancreatic cancer from five studies were pooled, showed that weight loss at diagnosis had a significantly poorer prognosis than the others, especially older men (18). Pausch et al. (19) conducted a follow-up of 2,968 surgically treated pancreatic cancer patients and found that those with preoperative weight loss ≥10% had significantly elevated surgical and non-surgical complications (*P* < 0.03), along with notable increases in hospital mortality and 90-day mortality rates. The study also indicated that lean patients with a BMI <18.5 kg/m^2^ exhibited a significant increase in 90-day mortality, with a statistically significant difference (*P* = 0.048). On the contrary, patients with abundant abdominal and lumbar fat displayed significantly better long-term survival compared to lean patients, with *P* values of 0.047 and 0.022, respectively. However, a large number of studies have shown that BMI has no relation to the survival period of pancreatic cancer (8–10, 20).

Nutritional disturbances and cancer-related cachexia are frequently observed in individuals with pancreatic cancer, driven by a combination of metabolic and hormonal imbalances, tumor-induced anorexia, impaired nutrient intake and malabsorption, as well as heightened metabolic rates and systemic inflammation. These factors collectively contribute to a sustained negative energy balance, predominantly leading to the depletion of muscle mass and a decline in physical function (21). Weight loss is particularly pronounced, with average reductions exceeding 8% within 3 months and reaching over 12% 6 months before initial clinical assessment—an established hallmark of pancreatic cancer and often one of the earliest clinical indicators preceding diagnosis. Notably, despite the prevalence of these nutritional issues, the majority of patients do not receive formal nutritional counseling before beginning chemotherapy (22, 23). Malnutrition in cancer patients is primarily caused by increased inflammatory cytokine levels associated with the disease, metabolic imbalances, and poor nutrient consumption. This nutritional deficiency is frequently triggered by anorexia, which stems from both the tumor and the side effects of systemic treatments. The coexistence of malnutrition and loss of muscle mass is common in this population and markedly deteriorates clinical prognosis (24). The association between malnutrition and increased cancer-related mortality may be partially explained by a higher susceptibility to infections in these individuals. Evidence suggests that inadequate nutrition not only facilitates the development of infections but also impairs therapeutic efficacy (25). Numerous studies have indicated that poor nutritional status negatively impacts responses to chemotherapy and surgical interventions (26), while also contributing to immunosuppression (27). Additionally, low levels of lymphocytes have been correlated with malnutrition (28) and are significantly associated with poorer survival rates across various types of cancer (29–31).

The underlying mechanisms by which low body weight influences the prognosis of gastrointestinal cancers remain not fully understood (32). From a disease perspective, low BMI is a result of disease progression rather than a cause. Evidence suggests that weight loss is frequently linked to malnutrition in cancer patients. Cancer cells can accelerate the depletion of muscle mass, contributing to unintended weight reduction (33), and active nutritional support should be considered to improve their prognosis.

Prior research (34–36) has highlighted several modifiable factors that influence the likelihood of cancer recurrence and overall survival following treatment completion. Based on these findings, the American Cancer Society (ACS) has established specific recommendations for nutrition and physical activity tailored to cancer survivors. These guidelines emphasize maintaining a healthy body weight, participating in regular physical activity, adhering to a balanced diet, and abstaining from alcohol consumption (34). Studies indicate a positive correlation between adherence to these health-promoting behaviors and improved survival outcomes among cancer survivors (35, 36).

In summary, the present study has shown a significant association of BMI 4, BMI 5 and BMI 6 with PFS/OS of pancreatic cancer. Monitoring the weight change trajectory of patients with pancreatic cancer has significant clinical implications. Early identification of weight loss and active nutritional intervention may help break the cachexia cycle, improve treatment tolerance, enhance quality of life, and potentially prolong survival. Future research should further clarify the predictive value of dynamic patterns of weight change. For clinical practice, integrating regular nutritional screening and individualized nutritional support therapy into the comprehensive management of pancreatic cancer patients should become a standard treatment strategy. This is also in line with the overall recommendations of institutions such as the American Cancer Society (ACS) for cancer survivors, which emphasize maintaining a healthy weight. It should be noted that there are limitations to our study. First, as a single-center retrospective analysis, selection bias cannot be entirely excluded. Second, pre-diagnostic BMI data were collected retrospectively and may be subject to recall bias. Moreover, the sample size is relatively modest, and more detailed body-composition parameters (e.g., muscle mass, fat distribution) were not incorporated. Future prospective, multicenter studies should combine more precise body-composition analyses (such as CT-derived skeletal muscle index) and inflammatory biomarkers to further elucidate the underlying mechanisms linking weight change and prognosis.

Despite these limitations, this study provides the first systematic evidence that short-term BMI trajectories carry independent prognostic value in pancreatic cancer. Compared with static BMI measurements, dynamic monitoring of weight changes can identify high-risk patients with poor prognosis earlier and more sensitively, offering a strong rationale for timely nutritional and supportive interventions in clinical practice.

## Conclusions

This study confirms that short-term BMI trajectories before and after diagnosis, as well as early during treatment, are independent prognostic factors for both overall survival and progression-free survival in pancreatic cancer patients. Rapid weight loss in the year before diagnosis (a BMI decrease ≥1.3 kg/m^2^) and any degree of weight loss within 6 months after anti-tumor therapy (a BMI decrease >0.3 kg/m^2^) are both associated with significantly shortened survival. Particular attention should be paid to patients who are normal-weight at diagnosis but transition to a low-BMI category shortly after treatment, as they face the highest mortality risk.

Therefore, we recommend that regular, dynamic monitoring of weight and BMI be incorporated as an essential component of the comprehensive management of pancreatic cancer patients. Clinicians should maintain a high level of vigilance for patients exhibiting substantial short-term weight loss and promptly initiate individualized nutritional assessment and intervention strategies. Early recognition and active management of weight loss may help improve treatment tolerance, quality of life, and overall prognosis. Future research should aim to develop integrated prognostic models that combine dynamic BMI trajectories with other biomarkers, and to evaluate the precise impact of targeted nutritional support on survival outcomes in pancreatic cancer.

## Data Availability

The original contributions presented in the study are included in the article/Supplementary material, further inquiries can be directed to the corresponding author.
