# Supplementary material for: Short-term BMI trajectories as a prognostic predictor in patients with pancreatic cancer
Source: Front Nutr. 2026 Jan 30;12:1680626. doi: 10.3389/fnut.2025.1680626 (PMC12911407; doi:10.3389/fnut.2025.1680626)
Supplement: Supplementary file 1 [file Data_Sheet_1.docx]

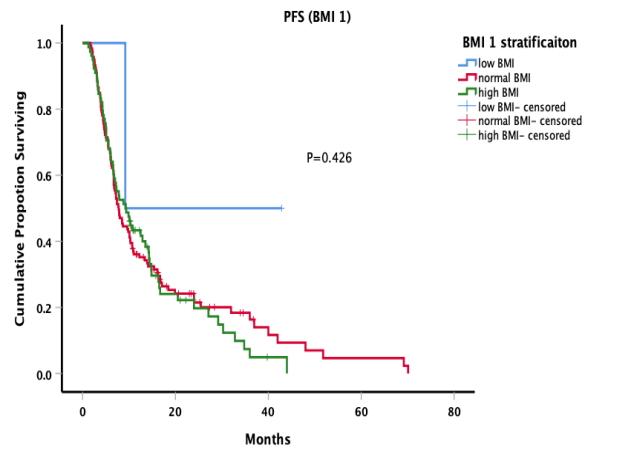

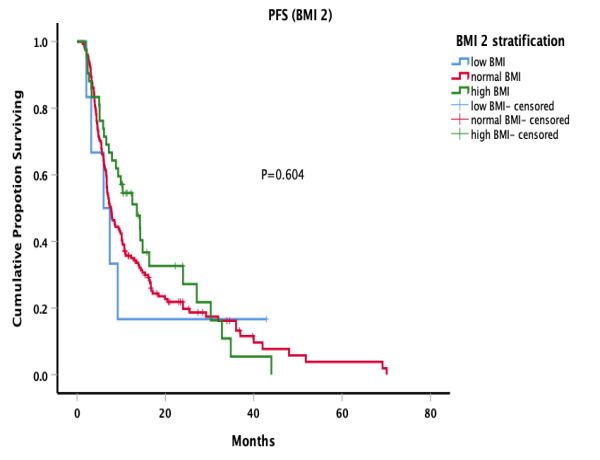


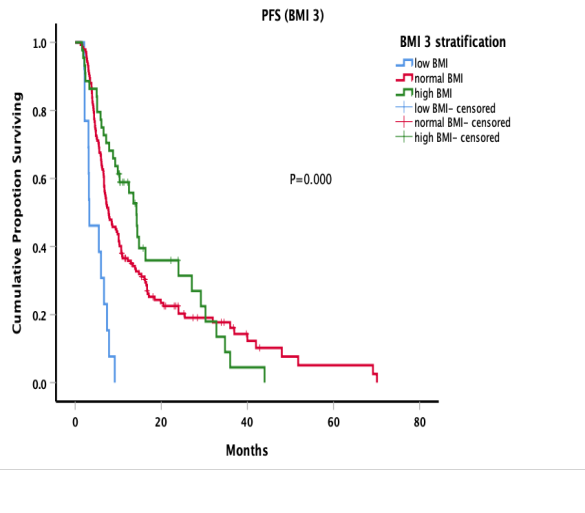

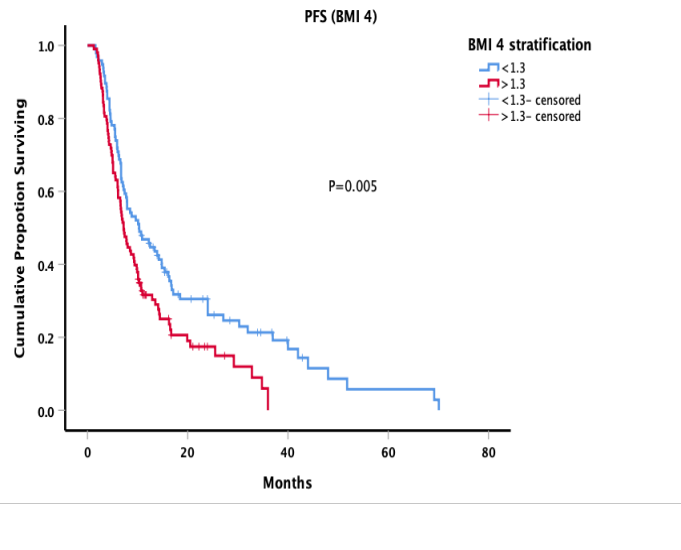


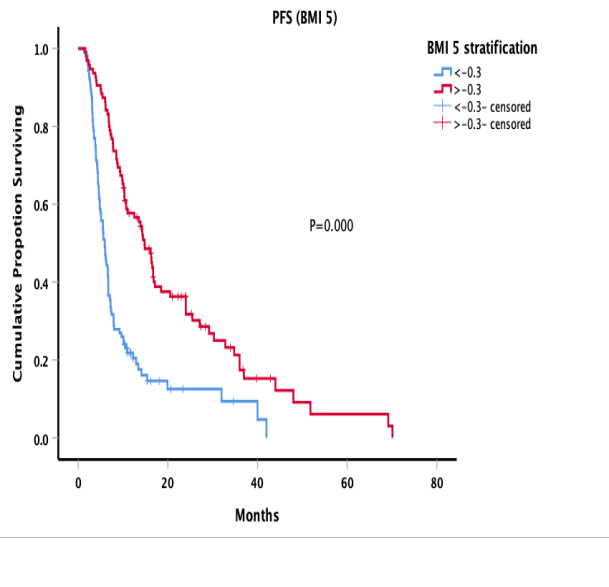

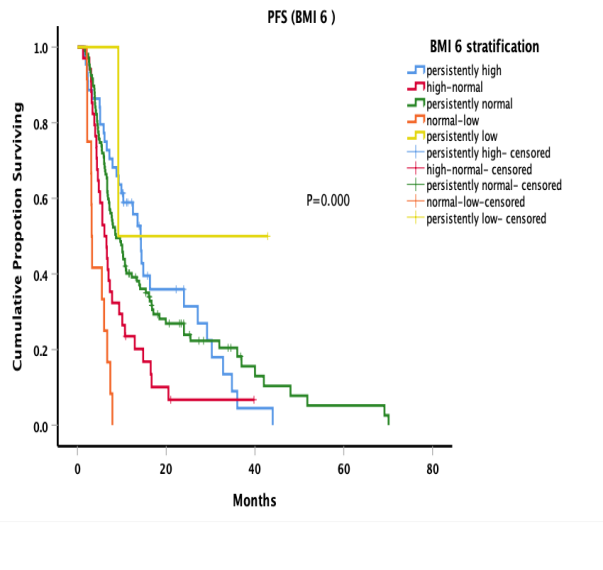


Supplementary figure 1: The relationship between BMI and PFS. There were significant differences in BMI 3, BMI 4 (BMI 1-BMI 2), BMI 5 (BMI 3-BMI 2) and BMI 6 (P<0.001, P=0.005, P<0.001, P<0.001), no significant differences were observed at BMI 1 and BM 2.


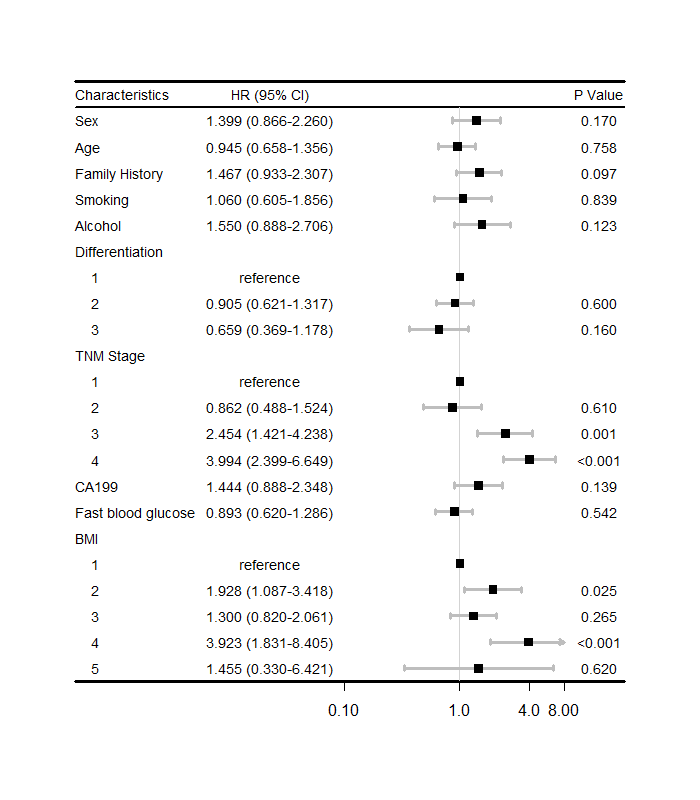


Supplementary figure 2. Forest plot of relationship between different factors and PFS. Abbreviations: age concentration is 60; differentiation: 1 is well, 2 is moderate, 3 is poor; CA199 concentration is 27 U/ml, fast blood glucose concentration is 7.0 mmol/L. BMI is BMI 6: 1. persistently high; 2. from high to normal (high- normal); 3. persistently normal; 4. from normal to low (normal-low); 5. persistently low.


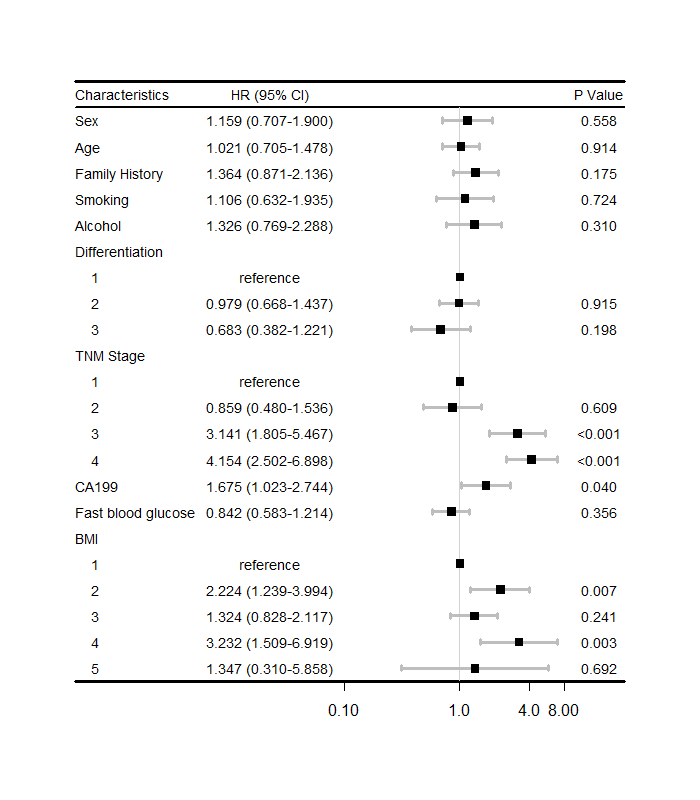


Supplementary figure 3. Forest plot of relationship between different factors and OS. Abbreviations: age concentration is 60; differentiation: 1 is well, 2 is moderate, 3 is poor; CA199 concentration is 27 U/ml, fast blood glucose concentration is 7.0 mmol/L. BMI is BMI 6: 1. persistently high; 2. from high to normal (high- normal); 3. persistently normal; 4. from normal to low (normal-low); 5. persistently low.


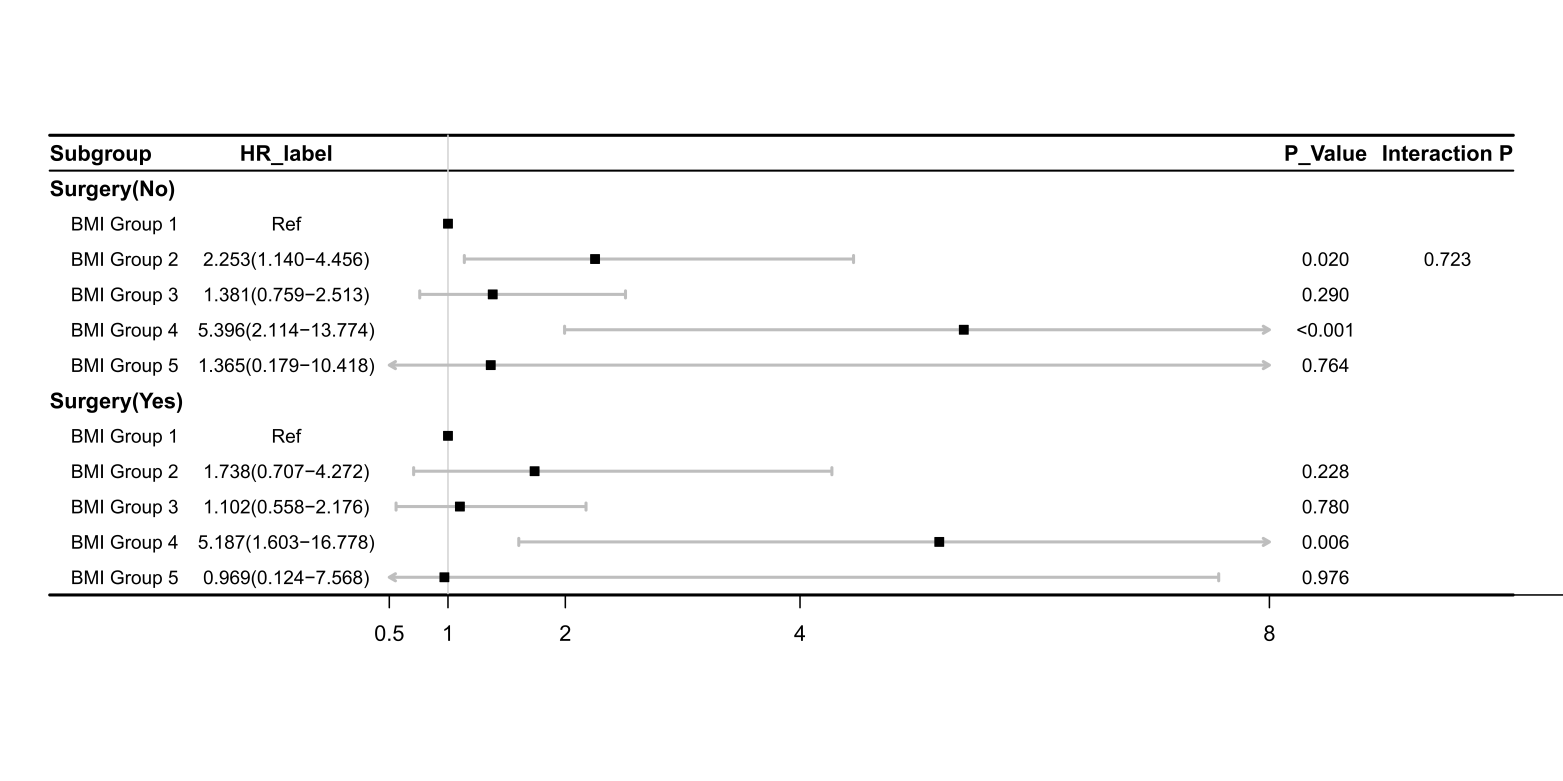


Supplementary figure 4. Forest plot of relationship between BMI 6 and PFS in different subgroup. Subgroup analyses were performed for surgery (yes or no), and no significant interactions were found in each subgroup (interaction P=0.723). Abbreviations: BMI is BMI 6: 1. persistently high; 2. from high to normal (high- normal); 3. persistently normal; 4. from normal to low (normal-low); 5. persistently low.


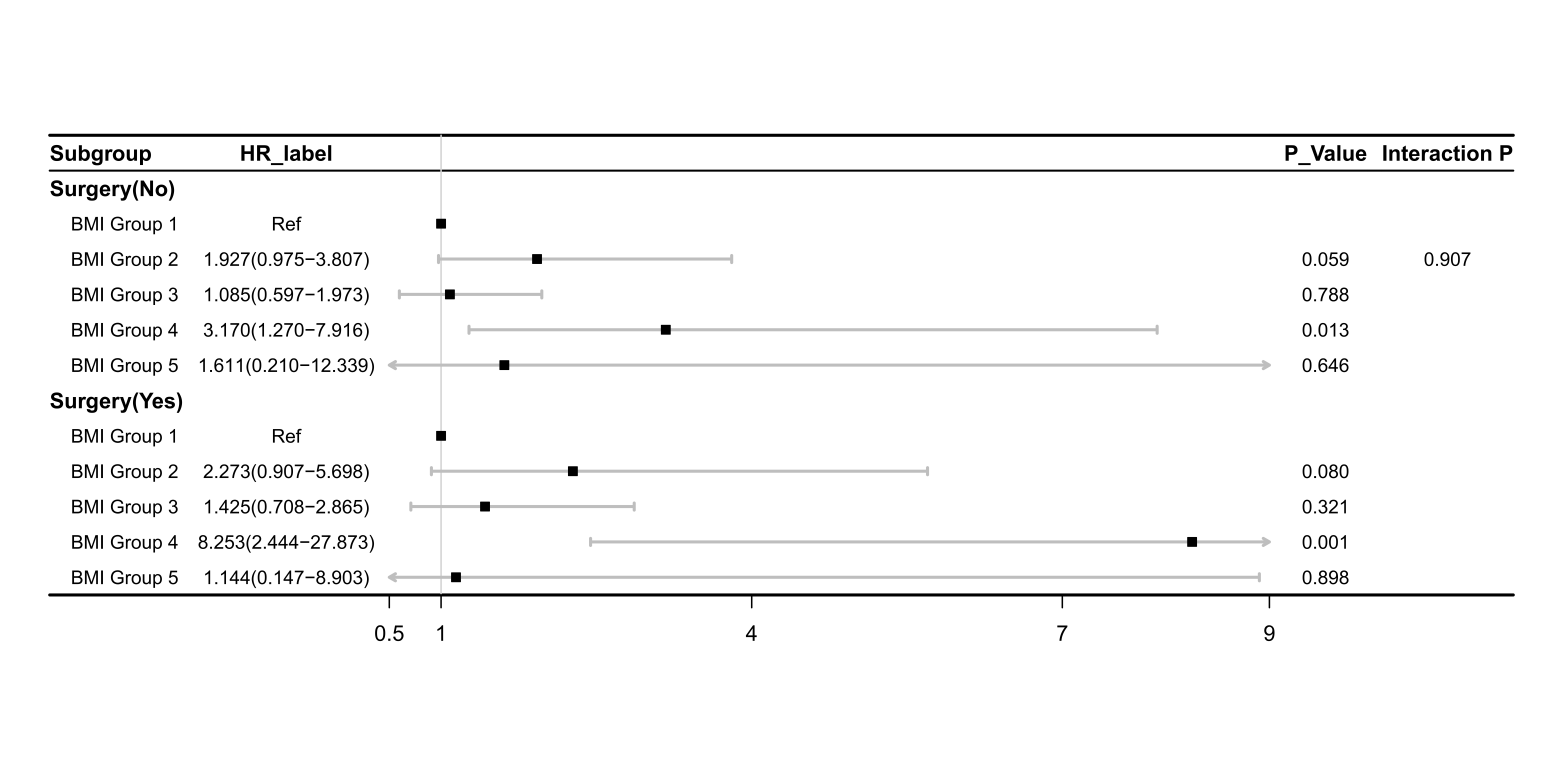


Supplementary figure 5. Forest plot of relationship between BMI 6 and OS in different subgroup. Subgroup analyses were performed for surgery (yes or no), and no significant interactions were found in each subgroup (interaction P=0.907). Abbreviations: BMI is BMI 6: 1. persistently high; 2. from high to normal (high- normal); 3. persistently normal; 4. from normal to low (normal-low); 5. persistently low.
